# Apolipoprotein E: from cardiovascular disease to neurodegenerative disorders

**DOI:** 10.1007/s00109-016-1427-y

**Published:** 2016-06-09

**Authors:** Robert W. Mahley

**Affiliations:** Gladstone Institute of Neurological Disease, 1650 Owens Street, San Francisco, CA 94158 USA; Departments of Pathology and Medicine, University of California, San Francisco, CA 94143 USA

**Keywords:** Alzheimer’s disease, ApoE, Atherosclerosis, Cholesterol, Small-molecule structure correctors

## Abstract

Apolipoprotein (apo) E was initially described as a lipid transport protein and major ligand for low density lipoprotein (LDL) receptors with a role in cholesterol metabolism and cardiovascular disease. It has since emerged as a major risk factor (causative gene) for Alzheimer’s disease and other neurodegenerative disorders. Detailed understanding of the structural features of the three isoforms (apoE2, apoE3, and apoE4), which differ by only a single amino acid interchange, has elucidated their unique functions. ApoE2 and apoE4 increase the risk for heart disease: apoE2 increases atherogenic lipoprotein levels (it binds poorly to LDL receptors), and apoE4 increases LDL levels (it binds preferentially to triglyceride-rich, very low density lipoproteins, leading to downregulation of LDL receptors). ApoE4 also increases the risk for neurodegenerative diseases, decreases their age of onset, or alters their progression. ApoE4 likely causes neurodegeneration secondary to its abnormal structure, caused by an interaction between its carboxyl- and amino-terminal domains, called domain interaction. When neurons are stressed or injured, they synthesize apoE to redistribute cholesterol for neuronal repair or remodeling. However, because of its altered structure, neuronal apoE4 undergoes neuron-specific proteolysis, generating neurotoxic fragments (12–29 kDa) that escape the secretory pathway and cause mitochondrial dysfunction and cytoskeletal alterations, including tau phosphorylation. ApoE4-associated pathology can be prevented by small-molecule structure correctors that block domain interaction by converting apoE4 to a molecule that resembles apoE3 both structurally and functionally. Structure correctors are a potential therapeutic approach to reduce apoE4 pathology in both cardiovascular and neurological disorders.

Early characterization of plasma lipoproteins highlighted the importance of their components, referred to as apolipoproteins, in controlling lipoprotein metabolism and cholesterol homeostasis [1]. Apolipoprotein (apo) E was late to be recognized as a critical protein constituent of lipoproteins [2]. First described in the early 1970s as a minor apolipoprotein in very low density lipoproteins (VLDL), apoE was subsequently identified as a major apolipoprotein in cholesterol-rich VLDL (β-VLDL) in cholesterol-fed animals [2–8] and found to be enriched in a subclass of high density lipoproteins (HDL; HDL_1_, HDL_c_) [8, 9]. Originally referred to as the arginine-rich apoprotein [2], the protein became known as apoE in 1982 [10].

ApoE has three common alleles encoded by the apoE gene on chromosome 19 [2]. These alleles occur at different frequencies in humans (ε2, 5–10 %; ε3, 65–70 %; and ε4, 15–20 %) and give rise to three homozygous (apoE2/2, apoE3/3, and apoE4/4) and three heterozygous (apoE3/2, apoE4/2, and apoE4/3) phenotypes. The structural basis for the three isoforms occurs through amino acid interchanges (single base changes in the apoE gene) at residues 112 and 158: apoE2 has cysteines at both sites, apoE4 has arginines, and apoE3 has cysteine (Cys)-112 and arginine (Arg)-158 [2, 11, 12]. All other animals, including the great apes, have a single isoform that has arginines at the residues equivalent to 112 and 158 [12, 13].

Plasma apoE is synthesized primarily by liver hepatocytes, which account for ~75 % of the body’s apoE production. In normolipidemic subjects, the plasma concentration of apoE is approximately 4–8 mg/dl. The second most common organ synthesizing apoE is the brain, where it is produced primarily by astrocytes, but also by oligodendrocytes, microglia, and neurons, especially injured or stressed neurons [see refs. 14–16 for more discussion]. In the brain, apoE is synthesized in situ and does not cross the blood brain barrier from the peripheral circulation [17]. Various cells throughout the body, including macrophages, also synthesize apoE [2, 11].

## Structure and function of apoE isoforms

**Fig. 1:**
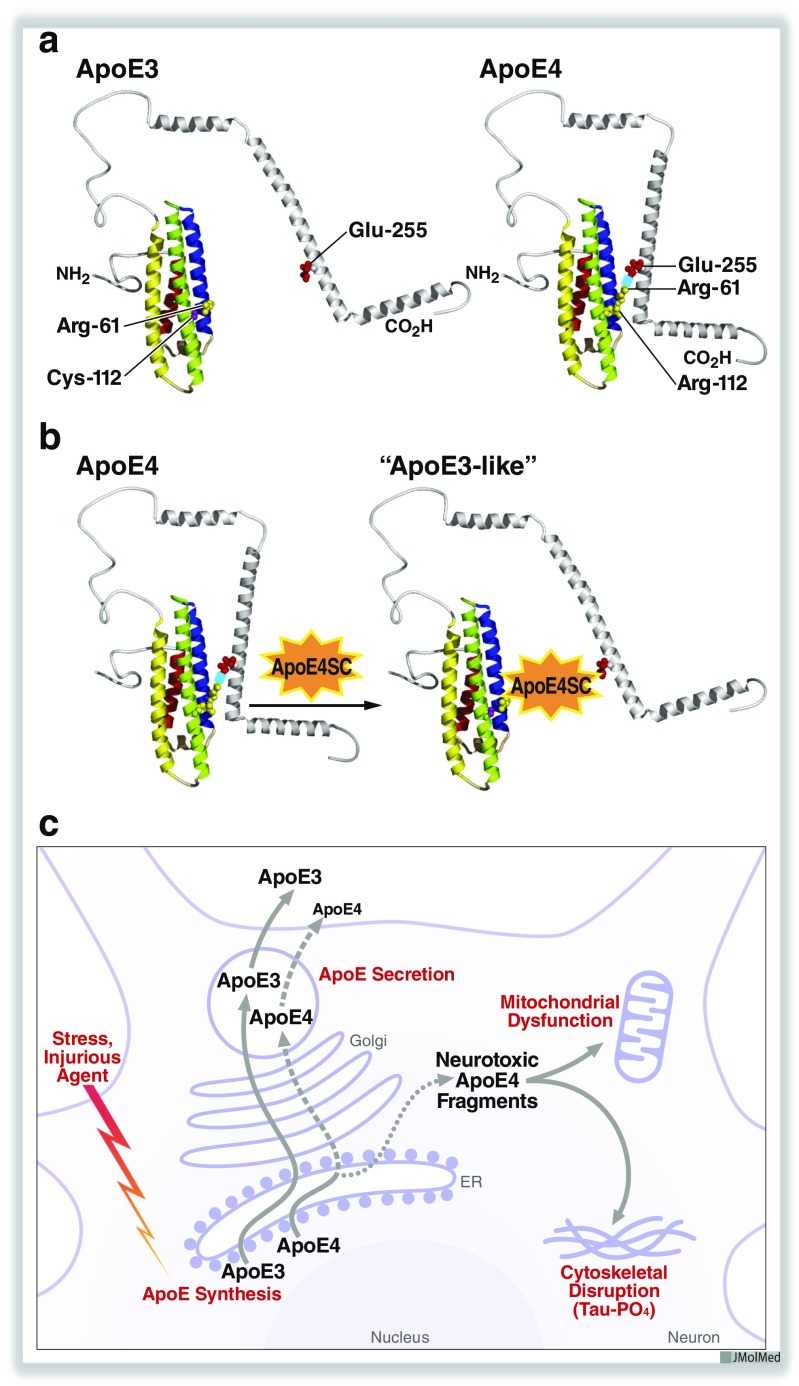
Structures of apoE3 and apoE4. **a** ApoE4 displays domain interaction caused by an ionic interaction between Arg-61 and Glu-255. This structural feature of apoE4 (apoE4 > apoE3 > apoE2) alters its function in cardiovascular and neurological disorders. **b** Small-molecule apoE4 structure correctors block domain interaction and convert apoE4 to an apoE3-like molecule structurally and functionally. **c** Injury of neurons induces apoE expression. When neurons are stressed or injured, they synthesize apoE to function in lipid redistribution for neuronal repair and remodeling. In this model, apoE4 is recognized as structurally abnormal and undergoes proteolytic cleavage, generating several neurotoxic fragments (12–29 kDa) that escape the secretory pathway, enter the cytosol, and cause mitochondrial dysfunction and tau phosphorylation (Tau-PO_4_), ultimately causing cell death. ApoE4SC, apoE4 structure corrector. Modified from ref. 38. Copyright 2012 American Chemical Society

ApoE, a 34-kDa protein of 299 amino acids with a single glycosylation site at threonine-194 [18], has two structural domains separated by a hinge region. The amino-terminal domain (amino acids 1–191) contains the low density lipoprotein (LDL) receptor binding region (amino acids 136–150) [11, 19, 20]. The carboxyl-terminal domain (amino acids ~225–299) contains the lipid binding region (amino acids ~240–260) [11, 13]. The tertiary structure of the amino-terminal domains of apoE4, apoE3, and apoE2, solved by x-ray crystallography, consists of four helices arranged in anti-parallel fashion [13]. The carboxyl-terminal domain has amphipathic α-helices that bind to lipids (Fig. 1a).

ApoE3 and apoE4 bind to the LDL receptor with similar affinity (~20-fold greater than that of apoB100, the other LDL receptor ligand). The key amino acids for receptor binding were identified by site-directed mutagenesis and by the existence of naturally occurring receptor-defective human mutations in type III hyperlipoproteinemic patients [11]. ApoE plays a major role in regulating cholesterol homeostasis by mediating the uptake of VLDL, intermediate density lipoproteins, and chylomicron remnants [2, 11, 12, 21]. ApoE2, however, defectively binds to the LDL receptor (~2 % of normal activity), because it has a cysteine at residue 158 rather than an arginine, as in apoE3 and apoE4. As shown by x-ray crystallography of the amino-terminal domain, Cys-158 prevents normal receptor binding by altering the conformation of the side chains in the critical basic residues in the 136–150 region. In apoE3, Arg-158 forms a salt bridge with aspartic acid–154; however, in apoE4, with Cys-158, this salt bridge is disrupted, and aspartic acid–154 interacts with Arg-150, altering the entire receptor binding region [11, 19, 20, 22].

ApoE4 has an arginine at residue 112 (Arg-112). This produces a property of apoE4 referred to as domain interaction, in which Arg-112 causes the side chain of Arg-61 to extend away from the amino-terminal domain [23, 24], enabling it to interact ionically with glutamic acid (Glu)–255 in the carboxyl-terminal domain (Fig. 1a). Domain interaction is less likely to occur in apoE3 or apoE2, because in these isoforms, with Cys-112, the side chain of Arg-61 is more tucked into the helical domain of the amino terminus [11, 24]. Domain interaction is unlikely to be an all-or-none property. Protein structure is dynamic, and it is likely that there is a gradient in the propensity of apoE to display domain interaction (apoE4 > apoE3 > apoE2).

### ApoE and cardiovascular disease

#### ApoE2

Because it binds defectively to LDL receptors, apoE2 homozygosity can precipitate type III hyperlipoproteinemia [2, 11, 21, 22]. This disorder occurs only when another condition—diabetes, obesity, hypothyroidism, or estrogen deficiency—leads to overproduction of VLDL or fewer LDL receptors, overwhelming the limited ability of apoE2 to mediate the clearance of triglyceride- and cholesterol-rich β-VLDL. Other dominant and recessive mutations in apoE that affect residues in or around the receptor binding region also cause type III hyperlipoproteinemia [2, 11, 22]. The defective receptor binding can precipitate hyperlipidemia in the context of other genetic or environmental factors and increase the risk for atherosclerosis, as the β-VLDL that accumulate in the plasma are highly atherogenic and cause cholesterol accumulation, especially in peripheral arteries.

#### ApoE4

ApoE4 increases plasma LDL levels and the risk for atherosclerosis [11, 12, 25]. The lipoprotein-binding preference of apoE4 to large (30–80 nm), triglyceride-rich VLDL, is associated with elevated LDL levels. (ApoE3 and apoE2 preferentially bind to small, 9–16-nm spherical HDL particles enriched in phospholipids and surface proteins, primarily apoAI.) The enrichment of VLDL with apoE4 accelerates their clearance from the plasma by receptor-mediated endocytosis in the liver; as a result, LDL receptors are downregulated, and plasma LDL levels increase [26–28]. Hepatic clearance of apoE-enriched lipoproteins involves LDL receptors and LDL receptor-related proteins (members of the LDL receptor family) and heparan sulfate proteoglycans—all of which interact with high affinity with apoE [for review, see refs. 29, 30].

How does the amino acid difference at residue 112 in the amino-terminal domain—arginine in apoE4 and cysteine in apoE3 and apoE2—alter the lipoprotein preference of apoE4 when lipid binding is mediated by residues 240–260 in the carboxyl-terminal domain? The reason is the structural effect of domain interaction. When domain interaction is disrupted with small molecules that prevent the Arg-61–Glu-255 ionic interaction or by site-directed mutagenesis (Arg-61 to threonine), apoE4’s binding preference shifts from VLDL to smaller, phospholipid-rich HDL [23, 24, 31]. In fact, apoE4 domain interaction is associated with decreased phospholipid-binding capacity [32]. Domain interaction may stabilize an extended helical structure involving the amino- and carboxyl-terminal domains of apoE4 [33–35], allowing it to interact with larger VLDL and accommodating interaction with lipoprotein particles with less curvature.

### ApoE4 and neurological disease

ApoE4 is the major genetic risk factor (or causative gene) for Alzheimer’s disease (AD) and other neurological disorders, including poor clinical outcomes after traumatic brain injury, or stroke, frontotemporal dementia, Down syndrome, certain patients with Parkinson’s disease, and Lewy body disease [for review, see refs. 12, 36–38]. ApoE4 dramatically affects AD, and 65–80 % of all AD patients carry at least one apoE4 allele. ApoE4 increases the risk of developing AD by 4-fold (one allele) to 14-fold (two alleles) compared with apoE3/3 homozygosity, and it decreases the age of onset by 8 years for each apoE4 allele (onset by mid-1960s with two alleles). Importantly, apoE4 alleles are not rare: ~25 % of people worldwide have at least one apoE4 allele.

Multiple factors acting through various pathways cause cognitive decline and neurodegeneration. Several mechanisms have been proposed, but the so-called amyloid hypothesis has received the most attention [for review, see refs. 39–41]. Rare mutations in the gene for the human amyloid precursor protein and the enzymes (secretases) that generate elevated levels of the amyloid beta (Aβ) peptide are related to the development of early-onset AD [42–44]. These genetic variants explain 1–2 % of AD cases; the majority of AD is sporadic. Mouse models of AD in which these variants are markedly overexpressed display some of the pathological features of AD, including increased Aβ and amyloid plaques, augmented tau phosphorylation, loss of synaptic connections, and impaired learning and memory, but not the significant neurodegeneration seen in AD [37, 43, 45, 46]. Aβ accumulation is considered neurotoxic.

#### Mechanisms for apoE4’s involvement in the amyloid pathway

There is no consensus concerning the mechanisms by which apoE4 affects the amyloid pathway [39–41]. Several lines of evidence suggest that apoE4 accelerates Aβ deposition to form amyloid plaques [47]. Others suggest that apoE4 is deficient in Aβ clearance [48, 49]. Some studies show that apoE3 binds Aβ to a greater extent than apoE4 and mediates the uptake and degradation of Aβ by receptor-mediated endocytosis in the brain, whereas apoE4 is less efficient in Aβ clearance and is associated with increased Aβ levels [39–41]. Others have shown that Aβ does not interact with apoE at all and suggest that, by an unknown mechanism, apoE4 competes with an Aβ clearance mechanism and thus increases Aβ levels [50].

Lipidation of apoE-containing lipoproteins in the brain has been postulated to modulate amyloid deposition and Aβ clearance by delivering Aβ to microglia and astrocytes for degradation or to the blood brain barrier for transport out of the brain [39, 40, 51, 52]. ApoE mediates lipoprotein interaction with various members of the LDL receptor family involved in these processes [39, 40]. Lipidation of apoE in the brain occurs through the activity of specific ATP-binding cassette transporters (ABC), including ABCA1 [53] and possibly ABCA7 [54], ABCG1 [55, 56], and ABCG4 [56], which are expressed in the brain. ApoE4 levels [57, 58] and the degree of apoE4 lipidation [32] are decreased in the brain compared with apoE3 and apoE2, and this has been correlated with AD risk. Overexpression of ABCA1 in an AD mouse model reduces amyloid deposition and improves Aβ clearance [59]. Alternatively, decreased ABCA1 expression reduced apoE levels and lipidation and increased amyloid deposition [60, 61]. However, the importance of ABCA1 activity in the human brain remains unknown, and existing data are conflicting. Recently, a population-based study demonstrated that a loss-of-function mutation in ABCA1 was associated with low plasma levels of apoE (no data on brain apoE levels) and increased risk of AD [62]. On the other hand, lipidation of apoE- and apoAI-containing lipoproteins in the brain may act independently of Aβ metabolism by protecting the integrity of the blood brain barrier and maintaining normal cerebrovascular function [63]. Whether modulation of brain lipoprotein lipidation represents a viable therapeutic approach remains to be determined.

Regardless of the reason, apoE4 carriers have increased numbers of amyloid plaques in their brains [37, 39, 64]. However, the plaque accumulation commences early in life, before cognitive impairment is evident, and does not entirely correlate with AD; about one-third of individuals with high levels of amyloid plaques are cognitively normal. AD in apoE4 carriers correlates better with the accumulation of phosphorylated tau in the hippocampus, which is critically important in cognition [65]. ApoE4 enhances tau phosphorylation and neurofibrillary tangle formation.

#### ApoE’s direct effects on neuropathology independent of Aβ

Studies from the Gladstone Institutes showed that apoE4 acts directly and independently of Aβ on a parallel path leading to neuropathology [12, 36–38]. By itself, apoE4 has many detrimental effects on neuronal cells in vitro and in vivo—including mitochondrial dysfunction due to decreased levels and activities of various electron transport enzymes and ATP synthase [66, 67]; increased tau phosphorylation and the formation of intracellular inclusions resembling neurofibrillary tangles [68, 69]; impairment in mitochondrial motility [67], intracellular trafficking of apoE [70], neurite outgrowth [67, 71], and synaptogenesis [72]; accelerated neuropathology involving loss of GABAergic hippocampal interneurons [69]; and impaired learning and memory in apoE4 mouse models [69, 73–75]. These detrimental effects are reversed when apoE4 domain interaction is blocked by site-directed mutagenesis (Arg-61 to threonine) or by small molecules that “correct” the structure of apoE4 [for review, see refs. 36–38].

#### Generation of apoE4 neurotoxic fragments

ApoE4 is highly susceptible to neuron-specific proteolysis, which generates 12–29-kDa neurotoxic fragments [68, 76, 77] (Fig. 1c). In response to injury or stress, neurons synthesize apoE, presumably to facilitate the rapid transport of cholesterol and other lipids for membrane repair and remodeling. Neuronal apoE synthesis is highly regulated by neuron-specific mRNA splicing [78]. We hypothesize that domain interaction causes apoE4 to be “sensed” by the cell as abnormal, targeting it for proteolytic cleavage in the endoplasmic reticulum or Golgi apparatus. ApoE3 is much less susceptible to this proteolysis, which does not occur in astrocytes or hepatocytes that also produce apoE. The initial cleavage removes the carboxyl-terminal 27–30 amino acids, generating a toxic 29-kDa fragment; subsequent proteolysis results in amino-terminal cleavage, generating 12–20-kDa fragments, some of which are also toxic.

These fragments escape the secretory pathway and enter the cytosol, where they stimulate tau phosphorylation and interact with mitochondria, causing mitochondrial dysfunction and neurodegeneration [36–38] (Fig. 1c). Fragments containing the receptor binding region (residues 136–150) and the lipid binding region (residues 240–260) are the minimal structure of apoE responsible for translocation, mitochondrial localization, and neurotoxicity [79]. The receptor binding region behaves like a protein translocation domain, as in viral proteins (region of a protein enriched in arginines and lysines that facilitates translocation of a protein across a membrane). The hydrophobicity of the lipid binding region mediates the interaction of the fragments with mitochondria and subsequent neurotoxicity. The critical residues in these regions of apoE have been identified by site-directed mutagenesis. In the brain, the apoE fragments are much more abundant in patients with AD than in age-matched, nondemented controls [76, 80]. The fragments also occur in the brains of mice expressing apoE4 in neurons.

#### Identifying structure correctors to block apoE4 domain interaction

Small-molecule structure correctors that block the ionic interaction of Arg-61 and Glu-255 in apoE4 (Fig. 1b) have been identified with a cellular fluorescence resonance energy transfer (FRET) assay in which the amino-terminal domain of apoE4 is labeled with green fluorescent protein and the carboxyl-terminal domain is labeled with *Escherichia coli* dihydrofolate reductase. The FRET assay measures the ability of an active structure corrector to prevent the FRET emission signal that occurs in apoE4 when the amino- and carboxyl-terminal domains are in close proximity [67]. Testing apoE4 structure correctors with this assay revealed downstream functional effects in neurons. For example, active structure correctors restore mitochondrial cytochrome c oxidase levels, which are depleted in apoE4-expressing cultured neurons [67]. Structure–activity relationships have been established for such compounds at low nanomolar levels.

#### Retarding apoE4-associated neuropathology in vivo in mice

Overexpression of apoE4 in transgenic mice or expression of apoE4 in targeted-replacement mice causes central nervous system pathology, including increased tau phosphorylation, loss of interneurons, and impaired learning and memory [69, 73–75]. A prototypical apoE4 structure corrector, PY-101, has been used in the apoE4 transgenic mice as a proof-of-concept that blocking apoE4 domain interaction can reverse the detrimental effects associated with apoE4 [38]. After intraperitoneal or subcutaneous administration of PY-101 (30–50 mg/kg body weight for 10 consecutive days), apoE4 fragment levels in the brain and hippocampus decreased by 20–25 %, and mitochondrial cytochrome c oxidase levels increased by 50–55 %. Preclinical studies are in process, and potent small molecules with drug-like properties have been identified. Thus, apoE4 is a promising drug target for apoE4-associated neuropathology in AD and other disorders. As an additive or ancillary therapy, apoE structure correctors might also help reduce plasma LDL levels and risk for coronary artery disease.
